# Dominant negative variants in *KIF5B* cause osteogenesis imperfecta via down regulation of mTOR signaling

**DOI:** 10.1371/journal.pgen.1011005

**Published:** 2023-11-07

**Authors:** Ronit Marom, Bo Zhang, Megan E. Washington, I-Wen Song, Lindsay C. Burrage, Vittoria C. Rossi, Ava S. Berrier, Anika Lindsey, Jacob Lesinski, Michael L. Nonet, Jian Chen, Dustin Baldridge, Gary A. Silverman, V. Reid Sutton, Jill A. Rosenfeld, Alyssa A. Tran, M. John Hicks, David R. Murdock, Hongzheng Dai, MaryAnn Weis, Shalini N. Jhangiani, Donna M. Muzny, Richard A. Gibbs, Richard Caswell, Carrie Pottinger, Deirdre Cilliers, Karen Stals, Undiagnosed Diseases Network, David Eyre, Deborah Krakow, Tim Schedl, Stephen C. Pak, Brendan H. Lee

**Affiliations:** 1 Department of Molecular and Human Genetics, Baylor College of Medicine, Houston, Texas, United States of America; 2 Texas Children’s Hospital, Houston, Texas, United States of America; 3 Department of Pediatrics, Washington University School of Medicine, St Louis, Missouri, United States of America; 4 Department of Neuroscience, Washington University School of Medicine, St Louis, Missouri, United States of America; 5 Department of Genetics, Washington University School of Medicine, St Louis, Missouri, United States of America; 6 Department of Pathology & Immunology, Baylor College of Medicine, Houston, Texas, United States of America; 7 Department of Orthopaedics and Sports Medicine, University of Washington, Seattle, Washington, United States of America; 8 Exeter Genomics Laboratory, Royal Devon University Healthcare NHS Foundation Trust, Exeter, United Kingdom; 9 All Wales Medical Genomics Service, Wrexham Maelor Hospital, Wrexham, UK; 10 Oxford Centre for Genomic Medicine, Oxford University Hospitals NHS Foundation Trust, Oxford, United Kingdom; 11 Human Genetics, Obstetrics & Gynecology, Orthopedic Surgery, University of California, Los Angeles, California, United States of America; HudsonAlpha Institute for Biotechnology, UNITED STATES

## Abstract

**Background:**

Kinesin motor proteins transport intracellular cargo, including mRNA, proteins, and organelles. Pathogenic variants in kinesin-related genes have been implicated in neurodevelopmental disorders and skeletal dysplasias. We identified *de novo*, heterozygous variants in *KIF5B*, encoding a kinesin-1 subunit, in four individuals with osteogenesis imperfecta. The variants cluster within the highly conserved kinesin motor domain and are predicted to interfere with nucleotide binding, although the mechanistic consequences on cell signaling and function are unknown.

**Methods:**

To understand the *in vivo* genetic mechanism of *KIF5B* variants, we modeled the p.Thr87Ile variant that was found in two patients in the *C*. *elegans* ortholog, *unc-116*, at the corresponding position (Thr90Ile) by CRISPR/Cas9 editing and performed functional analysis. Next, we studied the cellular and molecular consequences of the recurrent p.Thr87Ile variant by microscopy, RNA and protein analysis in NIH3T3 cells, primary human fibroblasts and bone biopsy.

**Results:**

*C*. *elegans* heterozygous for the *unc-116* Thr90Ile variant displayed abnormal body length and motility phenotypes that were suppressed by additional copies of the wild type allele, consistent with a dominant negative mechanism. Time-lapse imaging of GFP-tagged mitochondria showed defective mitochondria transport in *unc-116* Thr90Ile neurons providing strong evidence for disrupted kinesin motor function. Microscopy studies in human cells showed dilated endoplasmic reticulum, multiple intracellular vacuoles, and abnormal distribution of the Golgi complex, supporting an intracellular trafficking defect. RNA sequencing, proteomic analysis, and bone immunohistochemistry demonstrated down regulation of the mTOR signaling pathway that was partially rescued with leucine supplementation in patient cells.

**Conclusion:**

We report dominant negative variants in the *KIF5B* kinesin motor domain in individuals with osteogenesis imperfecta. This study expands the spectrum of kinesin-related disorders and identifies dysregulated signaling targets for *KIF5B* in skeletal development.

## Author summary

Kinesin-related gene defects are associated with multi-systemic disorders, also known as “Kinesinopathies”. Heterozygous variants in *KIF5B*, the gene encoding the Kinesin-1 heavy chain, have been implicated in neurodevelopmental delay, skeletal dysplasia, myopathy, and dysmorphic features. We identified *de novo*, heterozygous missense variants in *KIF5B* kinesin motor domain in four individuals with osteogenesis imperfecta. Findings in animal model and cell studies point to a dominant negative mechanism, an intracellular trafficking defect, and alteration of mTOR signaling. The study identifies *KIF5B* variants as a novel cause for osteogenesis imperfecta and suggests that mTOR signaling may be potentially targeted for future therapy in *KIF5B*-related disorder.

## Introduction

Kinesin superfamily of motor proteins (KIFs) play an essential role in fundamental cellular processes, including endocytosis, cell cycle, autophagy and ciliogenesis [1–3]. Within this family, kinesin-1 utilizes ATP to transport cargo, such as mRNA, secretory vesicles, mitochondria, and lysosomes along oriented microtubule tracks [4–7]. Kinesin-1 is a tetramer composed of two microtubule-associated heavy chains and two cargo-binding light chain molecules [8,9]. The kinesin-1 heavy chain is encoded by either of three *KIF5* genes: *KIF5B*, which is ubiquitously expressed, or its neuron-specific heavy chain isoforms *KIF5A* and *KIF5C* [3,8]. Homozygous knockout of *Kif5b* in mice (*Kif5b*^*-/-*^) results in severe growth retardation and embryonic lethality at E. 9.5–11.5 [10]. Conditional deletion of the murine *Kif5b* in pancreatic beta cells in *Kif5b*^*fl/-*^;RIP2-Cre mice is associated with a defect in insulin secretion, decreased islet size, and diabetes [11]. *Kif5b*^*fl/-*^; Pax2-Cre mice that have a genetic deletion of *Kif5b* in skeletal muscle exhibit impaired myofibril assembly and muscular dystrophy [12]. Genetic deletion of *Kif5b* in the chondrocyte lineage via Col2a1-Cre was associated with a chondrocyte differentiation defect and disruption of the growth plate ultrastructure, leading to growth retardation [13]. Consistent with these findings, *kif5b* loss of function in zebrafish resulted in abnormal craniofacial development, with deficits in muscle development, chondrogenesis and skeletal mineralization [14]. While these studies highlight the critical role of *KIF5B* in development across tissues and species, downstream signaling and cellular consequences are poorly understood.

Pathogenic variants in KIF-related genes, also known as “kinesinopathies” [15], have been associated with variable human phenotypes, including neurodevelopmental disorders and skeletal dysplasias [16–22]. Itay *et al*. reported heterozygous missense variants in *KIF5B* in individuals that were clinically diagnosed with kyphomelic dysplasia and presented with short stature, osteoporosis, fractures, bone deformities, and variable developmental delay [23]. Flex *et al*. reported heterozygous pathogenic variants in *KIF5B* in individuals with developmental delay, skeletal myopathy, and adult-onset cardiomyopathy but no features of skeletal dysplasia [24].

Here, we identified heterozygous, *de novo* variants in *KIF5B* in four unrelated individuals with osteogenesis imperfecta, including a recurrent variant p.Thr87Ile that resides in the highly conserved nucleotide-binding P-loop domain. The recurrent variant p.Thr87Ile was modeled *in vivo* in *C*. *elegans* and studied *in vitro* to assess the cellular and molecular alterations downstream of *KIF5B* variants. This study expands the clinical spectrum of *KIF5B*-related disorder and informs on the mechanism of pathogenic variants in kinesin-1 in skeletal dysplasia.

## Results

### *De novo*, heterozygous variants in *KIF5B* were identified in individuals with osteogenesis imperfecta

**Fig 1:**
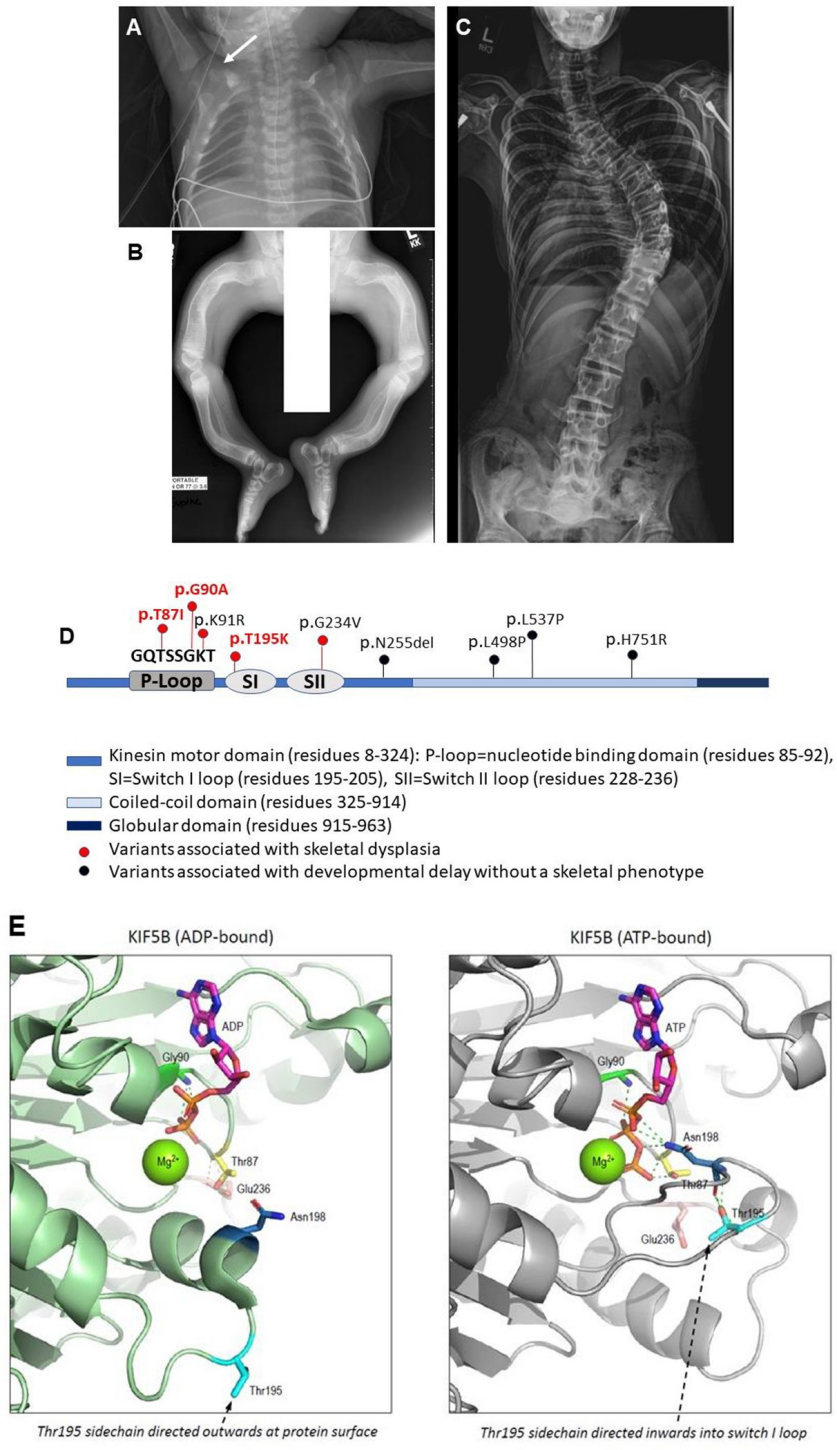
Recurrent heterozygous variants in *KIF5B* cause osteogenesis imperfecta. (A-C) Skeletal radiographs of Proband 1. (A) Osteopenia with gracile ribs and fracture of clavicle (arrow) at age 1 week; (B) Healing fractures and bowing of long bones, early childhood; (C) Scoliosis, adolescent years. (D) Graphic illustration of *KIF5B* gene, highlighting pathogenic variants reported here (red, bold) and elsewhere [23,24,83], within functional domains. In red: Pathogenic variants associated with skeletal dysplasia (within the kinesin motor domain, the P-loop and the switch loops create and stabilize the nucleotide binding pocket). The amino acids sequence within the P loop domain, outlined in the figure, is highly conserved between species. In black: Pathogenic variants outside the P loop and switch loop domains are not associated with a skeletal phenotype. (E) KIF5B nucleotide binding and potential mechanism of variants reported here: In the ADP-bound form of KIF5B (left), the backbone of Gly90 (bright green) makes hydrogen bonds (green broken lines) with ADP, while the backbone of Thr87 (yellow) makes hydrogen bonds (yellow broken lines) with Glu236. Note that, in this form, Thr195 (cyan) is distant from the bound nucleotide with the sidechain directed outward of the protein surface. In the ATP-bound form of KIF5B (right), Gly90 bonding to ATP is retained while the movement of the switch I loop brings Thr195 and Asn198 (blue) sidechains into proximity with the nucleotide, allowing multiple potential hydrogen bonds with phosphate groups (green broken lines) and stabilization of the conformational switch.

We performed a clinical and molecular study in four individuals with an overlapping phenotype characterized by hypomineralization of bones, multiple fractures, and skeletal deformities (**Fig 1A–1C**). Exome sequencing identified *de novo*, heterozygous variants in *KIF5B* that reside within the kinesin motor domain (**Fig 1D** and **Table 1**). All variants identified substitute highly conserved amino acid residues and are predicted to destabilize the nucleotide binding pocket that is critical for the motor activity of the protein (**Fig 1E**).

**Table 1:** Clinical summary and *KIF5B* variants identified.

	Proband 1	Proband 2	Proband 3	Proband 4
***KIF5B* variant (NM_004521.2)**	c.260C>T p.Thr87Ile	c.260C>T p.Thr87Ile	c.269G>C p.Gly90Ala	c.584C>A p.Thr195Lys
**Inheritance**	*De novo*	*De novo*	*De novo*	*De novo*
**CADD score**	28.7	28.7	28.7	28.9
**Sex**	Female	Female	Female	Male
**Age at most recent evaluation (years)**	20	30	23+2/40 weeks gestation (POC)	20/40 weeks gestation (POC)
**Pregnancy & Birth**				
**IUGR/SGA**	Yes (BW 1673 grams)	Yes (BW 2353 grams)	Yes (BW 480 grams, 5%ile)	Yes (BW 265.3 grams, 7%ile)
**Gestational age at birth**	40 weeks	38 weeks	Pregnancy terminated at 23+2/40 weeks	Pregnancy terminated at 20/40 weeks
**Findings on prenatal ultrasound**	unknown	oligohydramnios	Short long bones with bowed femurs, *in utero* fractures	Abnormal ribs, short long bones, small chest size, hypomineralisation, tetralogy of Fallot, absent pulmonary valve, absent ductus venosus, micrognathia
**Growth**				
**Growth parameters on most recent evaluations (Z score or normal range)**	Height 134 cm (Z = -4.78) weight 28.2 kg (Z = -7.6)	Height 91.4 cm (Z = -10.8) weight 27.2 kg (Z = -9.2)	Weight 480 g (510 +/- 97 g) Crown-heel 25.5cm (29.1 +/- 2cm)	Weight 265.3g (307 +/- 47g) Crown-heel 20.5cm (24.4 +/- 1.8cm)
**Microcephaly**	Yes (FOC Z = -3.12)	No	No	No
**OI Features**				
**Osteopenia**	Yes	Yes	Yes	Yes
**Multiple fractures**	Yes	Yes	*In utero* bilateral fractures of femur, radius and ulna, and multiple rib fractures	No
**Bone deformities**	Yes	Yes	Yes (Angulation of humeri, right tibia and left fibula)	Yes (bent and short long bones, hypoplastic and unossified vertebrae and pelvic bones)
**Scoliosis**	Yes	Yes	No	No
**Dentinogenesis imperfecta**	Yes +Hypodontia, malocclusion	Yes	N/A	N/A
**Hearing loss**	Bilateral mild to moderate mixed hearing loss	Bilateral moderate to severe mixed hearing loss	N/A	N/A
**Pulmonary symptoms**	Restrictive lung disease (small chest)	Restrictive lung disease (small chest)	Slightly reduced lung weight/body weight ratio, normal lung histology	Small chest
**Other**				
**Dysmorphic craniofacial features**	Midface retrusion, malar flattening, Shallow orbits, mandibular prognathia	Relative macrocephaly, triangular shaped facies, proptosis, downslanting palpebral fissures, mildly low-set ears	Low-set ears, micrognathia, high arched palate, flattened nares	Hypertelorism, low-set ears, flat nasal bridge
**Congenital heart defect**	Moderate secundum ASD	No	No	Narrow pulmonary valve, dilated pulmonary artery branches, overriding aorta, absent ductus arteriosus
**Ophthalmology**	Drooping eyelids (Lt>Rt); bilateral lagophthalmos, hyperopia	Blue sclera, drooping eye lids	N/A	N/A
**Endocrine**	Delayed puberty	None	N/A	N/A
**Development & cognitive function**	Mild developmental delay (gross motor>fine>speech), normal cognitive function	Normal cognitive function	N/A	N/A
**Additional testing**				
**Chromosomal microarray analysis**	Normal	Not done	Normal	Normal
**Variants in OI-related genes**	Heterozygous VUS in *P3H1* (p.Asn491Ser), maternally inherited	*COL1A1* and *COL1A* negative OI gene panel (34 genes) negative	None	None
**Biochemical collagen analysis**	Normal migration (no overmodification) and normal 3-hydroxyproline analysis in fibroblasts	Not done	Not done	Not done

Abbreviations: ASD = atrial septal defect, BW = birth weight, IUGR = intrauterine growth restriction, POC = product of conception, N/A = not applicable, OI = osteogenesis imperfecta, SGA = small for gestational age at birth, VUS = variant of uncertain clinical significance.

Proband 1 is a 20-year-old female with a clinical diagnosis of osteogenesis imperfecta of moderate severity. She has a history of recurrent fractures with minor or no trauma, low bone mineral density, short stature, scoliosis and dentinogenesis imperfecta (**Fig 1A–1C**). In addition to the skeletal features, she has a history of bilateral mixed hearing loss, ptosis, an atrial septal defect, and a splenic cyst. She had mild developmental delay which resolved, and her academic performance in school was above average. Clinical genetic testing, including chromosome microarray analysis and OI gene panel, was non-diagnostic. Collagen analysis in skin fibroblasts showed normal gel migration and prolyl hydroxylation and ruled out a qualitative or quantitative collagen defect (**S1 Fig**). Research trio exome sequencing identified a heterozygous, *de novo* variant, c.260C>T, p.Thr87Ile (NM_004521.2) in *KIF5B* that was confirmed by Sanger sequencing. RNA sequencing in fibroblasts demonstrated that both the wild type and mutant alleles are expressed in the proband (sequencing detected the variant in 21/57, or 37% of the reads in proband’s fibroblasts). Proband 2 is a 30-year-old female with a clinical diagnosis of osteogenesis imperfecta. She has a history of low bone mineral density, multiple fractures, scoliosis and dentinogenesis imperfecta. She is also noted to have bilateral mixed hearing loss and ptosis. She had a typical development with no history of cognitive impairment. Research whole genome sequencing detected a heterozygous variant, c.260C>T, p.Thr87Ile (NM_004521.2) in *KIF5B*, same as found in proband 1. Probands 3 and 4 were both products of conception and pregnancies were terminated due to multiple congenital anomalies. The clinical findings, including hypomineralized bones, *in utero* fractures and bone deformities, were suspicious of osteogenesis imperfecta. Exome sequencing identified *de novo*, heterozygous variants: c.269G>C, p.Gly90Ala (proband 3), and c.584C>A, p.Thr195Lys (proband 4) in *KIF5B* (NM_004521.2). None of the *KIF5B* variants found in proband 1–4 were reported in gnomAD, a control population database [25]. The clinical and molecular findings in probands 1–4 are summarized in **Table 1**.

### The Thr90Ile variant is damaging to UNC-116 function, acting as a dominant negative allele

**Fig 2:**
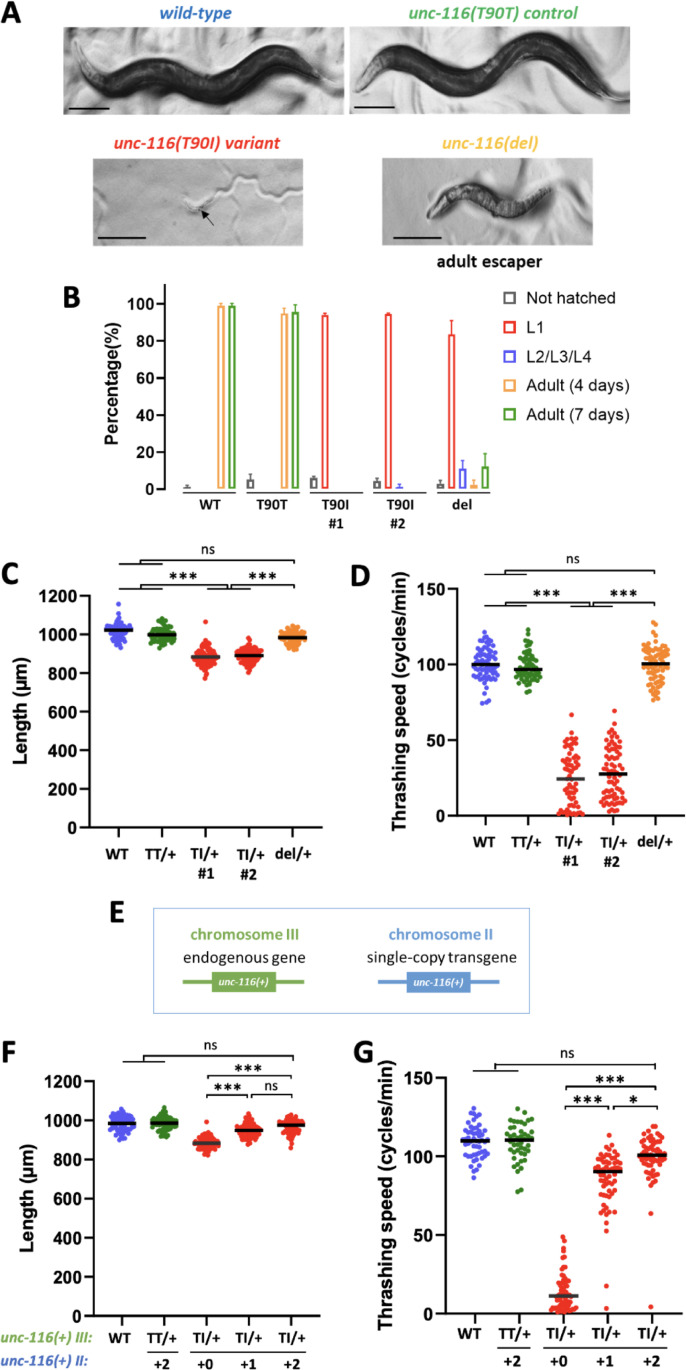
*unc-116(T90I)* is damaging and behaves as a dominant negative allele in *C*. *elegans*. (A) Images of wild-type (VC2010), *unc-116(T90T)* control edit, *unc-116(T90I)* variant edit, and *unc-116(del)* null worms. Homozygous *unc-116(T90I) and unc-116(del)* animals were L1-arrested and severely uncoordinated (Unc). *unc-116(del)* escaping adults were Unc and dumpy. Worms were grown for 7 days after egg-laying. Scale bar: 100 μm. (B) Developmental stages of animals scored 4 days after egg-laying (except for “Adult (7 days)” which were the total number of worms that grew to be adults after 7 days). *T90I#1* and *T90I#2* are two independent variant lines generated by CRISPR/Cas9 editing. Quantification of (C) body length and (D) thrashing speed of *unc-116(T90T#1) and unc-116(T90I#2)* heterozygotes. (E) Illustration showing the endogenous *unc-116* locus on chromosome III and the single-copy wildtype *unc-116* transgene integrated into a safe harbor locus on chromosome II. (F) Quantification of body length and (G) thrashing speed of *unc-116* T90T and T90I heterozygotes with extra copies of *unc-116* transgenes. Statistical analysis was performed using the Kruskall-Wallis multiple comparison tests. ns: not significant, *p<0.01, **p<0.001, ***p<0.0001.

To assess the functional impact of the p.Thr87Ile variant on KIF5B activity, we modeled the variant in the *C*. *elegans* ortholog, *unc-116*. The UNC-116 motor domain shares 71% amino acid identity (84% similarity) with KIF5B. Moreover, the proband variant resides in the P-loop of the ATP binding domain, which is 100% conserved in UNC-116 (**S2 Fig**). This allowed us to use CRISPR/Cas9 genome editing to precisely knock-in the Thr87Ile variant into the corresponding Thr90 residue in *unc-116*. As part of our editing strategy, we introduced synonymous changes to block Cas9 re-cleavage and generated a restriction enzyme site for allele genotyping. Therefore, as a control, we also generated the *unc-116(T90T)* edit that only contains the synonymous and restriction enzyme changes but not the variant edit. In addition, we obtained a deletion allele, *unc-116(del)*, derived from *unc-116(gk5722)* [26] for comparison. Animals homozygous for the *unc-116(T90T)* control developed normally and were indistinguishable from the wild-type VC2010 parental line (**Fig 2A and 2B**). In contrast, the *unc-116(T90I)* variant homozygotes arrested (and died) at the first larval (L1) stage (**Fig 2A and 2B**). Similarly, most of the *unc-116(del)* homozygotes arrested at the L1 stage, although approximately 10% of these animals eventually developed into adults (escaping adults) by 7 days after egg-lay (**Fig 2A and 2B**). These data indicated that the T90I variant is damaging to UNC-116 function and that the variant animals had a more severe defect than *unc-116(del)* null mutants.

Unlike the variant homozygotes, variant heterozygotes are viable and grow to be fertile adults. To determine whether the variant heterozygotes display dominant phenotypes, we quantified the animal’s body length and swimming speed using the WormLab 2019.1.1 (MBF Bioscience LLC). The body length of *unc-116(del)* heterozygotes was normal and not significantly different to that of *unc-116(T90T)* control heterozygotes (**Fig 2C**). However, the *unc-116(T90I)* variant heterozygotes had body lengths that were significantly shorter (**Fig 2C**). In terms of swimming, the *unc-116(T90T)* control and *unc-116(del)* heterozygotes thrashed similarly to the wild-type controls (**Fig 2D)**. However, the thrashing speed of the *unc-116(T90I)* variant heterozygotes was markedly slower than the controls (**Fig 2D** and **S1 and S2 Movies**). These results indicated that the *unc-116(T90I)* heterozygotes displayed dominant phenotypes not observed in deletion heterozygotes, suggesting that the T90I is a dominant negative or a hyperactive variant.

Next, we employed classic gene dosage studies [27,28] to better understand the nature of the *unc-116(T90I)* variant; if the variant gives rise to a dominant negative gene product, then adding wild type copies will suppress the phenotype, however, if the variant leads to a hyperactive gene product, then adding wild type copies will enhance the phenotype. The endogenous *unc-116* gene maps to chromosome III (**Fig 2E**, *green*). Using recombination-mediated cassette exchange (RMCE) [29,30], we inserted an additional copy of the wild-type *unc-116* gene into a safe harbor site on chromosome II (**Fig 2E**, *blue*). RNA-seq analysis confirmed that this extra copy was expressed at the same level as the endogenous gene (**S3A Fig**). Rescue studies demonstrated that two copies of *unc-116(+)* transgene were able to almost completely restore *unc-116(del)* mutants to wild-type level in body length and thrashing speed, indicating the transgene was fully functional (**S3B–S3C Fig**). Moreover, *unc-116(T90T)* control heterozygotes expressing two additional copies of the *unc-116(+)* transgene had the same body length and thrashed at the same speed as the wild-type animals indicating that expression of extra copies of the transgene did not adversely alter the phenotype (**Fig 2F and 2G**). In the *unc-116(T90I)* variant heterozygotes, one additional copy of *unc-116(+)* transgene partially suppressed the body length defect, while two additional copies fully restored the body length to wild-type levels (**Fig 2F**). Similar rescuing effects on thrashing were observed with additional copies of the *unc-116(+)* transgene (**Fig 2G**). In summary, our data indicates that three copies of the wild-type allele were required to suppress one copy of the variant allele, providing support that the *unc-116(T90I)* variant produces a dominant negative gene product that interferes with UNC-116 wild type function.

### The UNC-116 Thr90Ile variant impairs kinesin motor transport function

**Fig 3:**
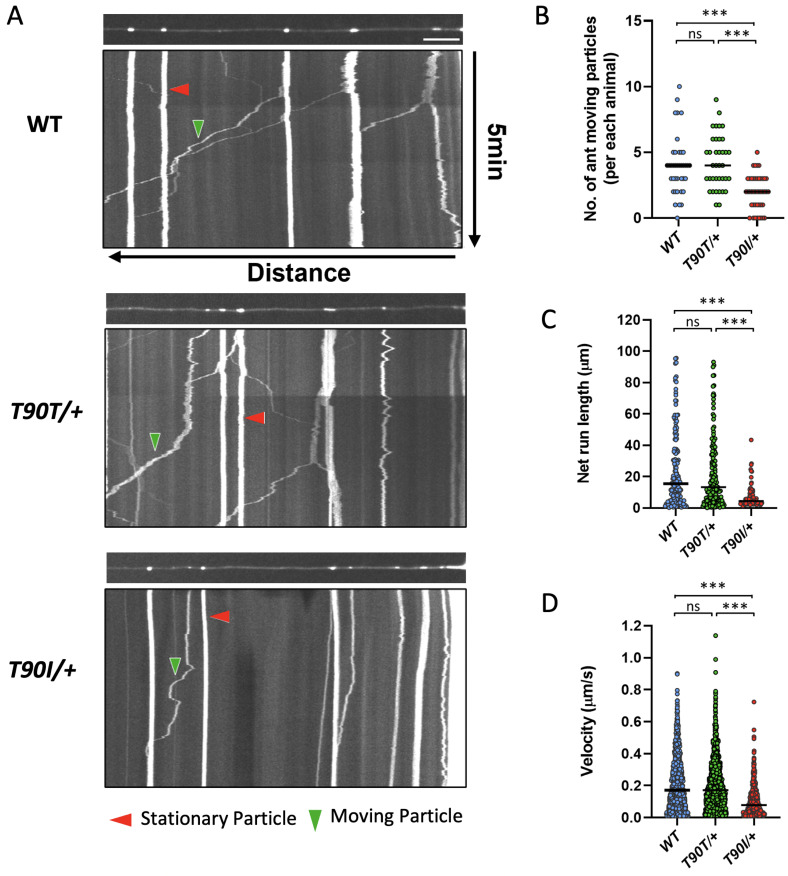
*unc-116(T90I)* disrupts kinesin-mediated transport of mitochondrial cargo along axonal microtubules. (A) Confocal images (upper) and kymographs (lower) of GFP-labeled mitochondria in ALM neurites of WT, *unc-116(T90T)* control heterozygotes, and *unc-116(T90I)* variant heterozygotes. Kymographs were generated from 5 min time lapse movies. Red arrowheads indicate stationary mitochondria. Green arrowheads indicate moving mitochondria. Scale bar: 10 μm. Quantification of (B) anterograde moving particle numbers, (C) net run length, and (D) combined segmental velocity. The mitochondrial marker used is *jsIs609 (mec-7p*::*mitoGFP)*. n ≥ 19 worms for each genotype. Kruskall-Wallis tests were used for multiple comparison. ns: not significant, ***p<0.0001.

To directly test the effect of the T90I variant on UNC-116 kinesin motor function, we visualized mitochondrial cargo transport along microtubules in the anterior neurite of the ALM touch neurons using the transgene, *jsIs609 (mec-7p*::*mitoGFP)* (**S1 Table**) [31]. We employed time-lapse confocal imaging to generate a 2D kymograph to track and quantify mitochondrial movement [32]. The kymographs identified several stationary (**Fig 3A**, vertical lines, *red arrowheads*) and moving mitochondria particles (**Fig 3A**, diagonal lines, *green arrowheads*). The number of moving particles was significantly reduced in *unc-116(T90I)* heterozygotes compared to wild-type and *unc-116(T90T)* heterozygote controls (**Fig 3B** and **S3 and S4 Movies**). Moreover, these particles traveled shorter distances (**Fig 3C**) and moved at a slower speed (**Fig 3D**) indicating that the kinesin motor activity was impaired in the UNC-116 T90I variant protein.

In summary, the experimental studies in *C*. *elegans* provide strong evidence for the UNC-116 T90I variant being damaging and causing defects in post-embryonic development, body length, locomotion, and kinesin motor function by acting as a dominant negative protein. To assess the effect of the *KIF5B* variant p.Thr87Ile on skeletal development, we used CRISPR/Cas9 editing to generate a *Kif5b*^*T87I*^ knock-in mouse model. However, no viable mice were identified by screening, likely due to early embryonic lethality.

### The *KIF5B* p.Thr87Ile variant is associated with an intracellular trafficking defect

**Fig 4:**
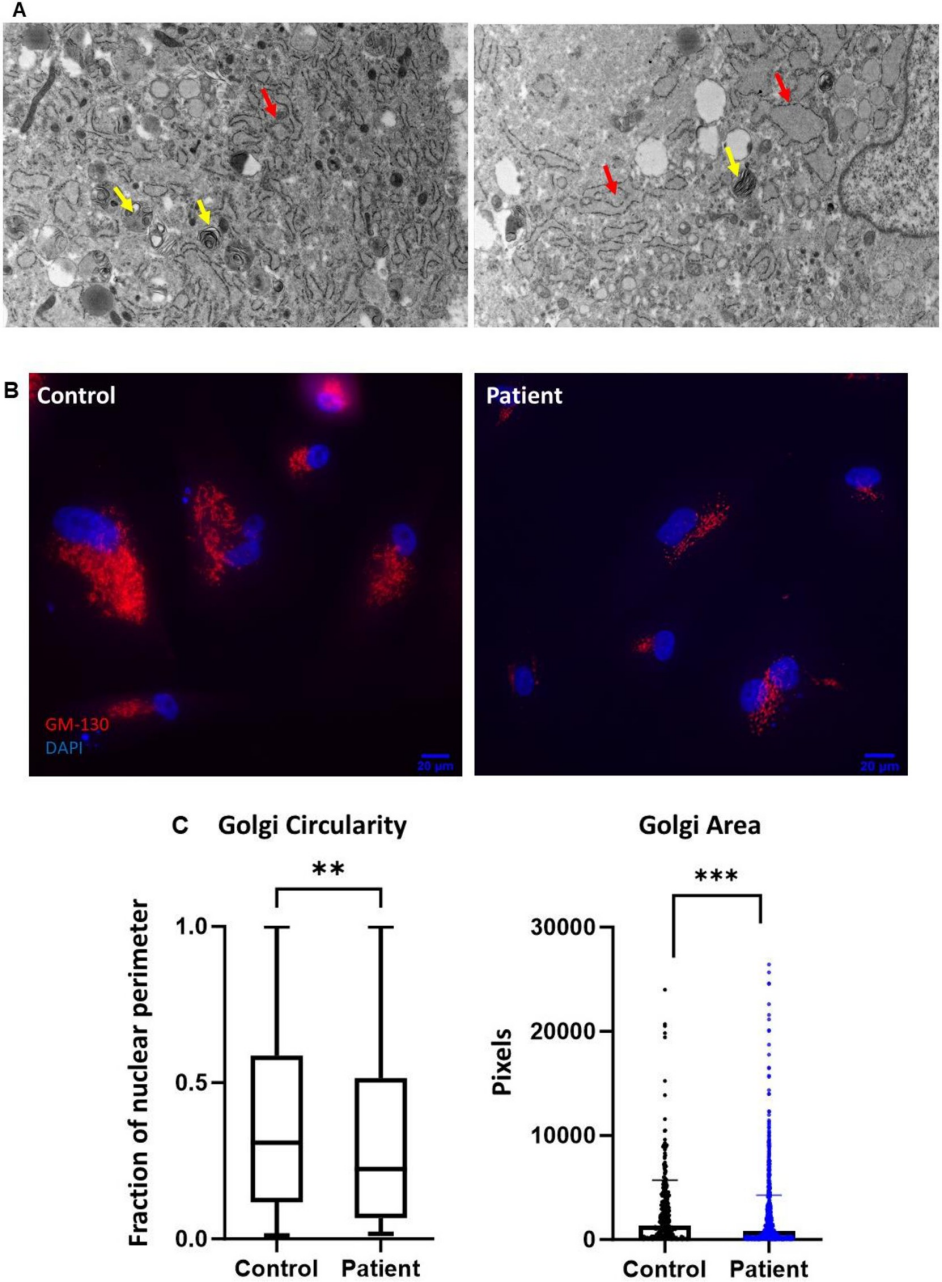
Dilated endoplasmic reticulum and altered Golgi distribution in patient cells suggest an intracellular trafficking defect. (A) Electron microscopy of proband 1 fibroblasts showing dilated endoplasmic reticulum (red arrows) and multiple vacuoles containing cell breakdown material (yellow arrows). (B) Representative image showing altered Golgi distribution in patient (proband 1) fibroblast cells (right) as compared to healthy control (left). (C) Quantification of the Golgi circularity and Golgi area in patient (proband 1) fibroblasts. Left: Decreased circularity in patient cells, measured as the fraction of nucleus surrounded by Golgi stacks, on a scale of 0 (highly polarized) to 1 (radially distributed). Right: Reduced Golgi area in patient cells, measured as the total area occupied by Golgi stacks. Graphs summarize multiple single-cell measurements (Mann-Whitney U test, n = 321 control fibroblasts, n = 887 patient fibroblasts, **P<0.001, ***p<0.0001).

To understand the cellular consequences of the recurrent *KIF5B* p.Thr87Ile variant we performed a microscopy study in primary patient fibroblasts. Electron microscopy on fibroblasts obtained from proband 1 showed prominent and dilated endoplasmic reticulum (ER) loops with abundant granular material, and multiple vacuoles containing cell breakdown products (**Fig 4A**). This pattern suggests a disruption of intracellular trafficking that is expected to affect the organization and function of the ER-Golgi complex. Consistently, immunofluorescent staining for the cis-Golgi matrix protein GM-130 in proband 1 fibroblasts showed altered Golgi distribution, with collapsed Golgi stacks (**Fig 4B**). Quantification in cells labeled with CellLight Golgi-GFP reagent showed decreased Golgi area as compared to control fibroblasts (**Fig 4C**).

### Primary cilia length is altered in cells expressing the *KIF5B* p.Thr87Ile variant

**Fig 5:**
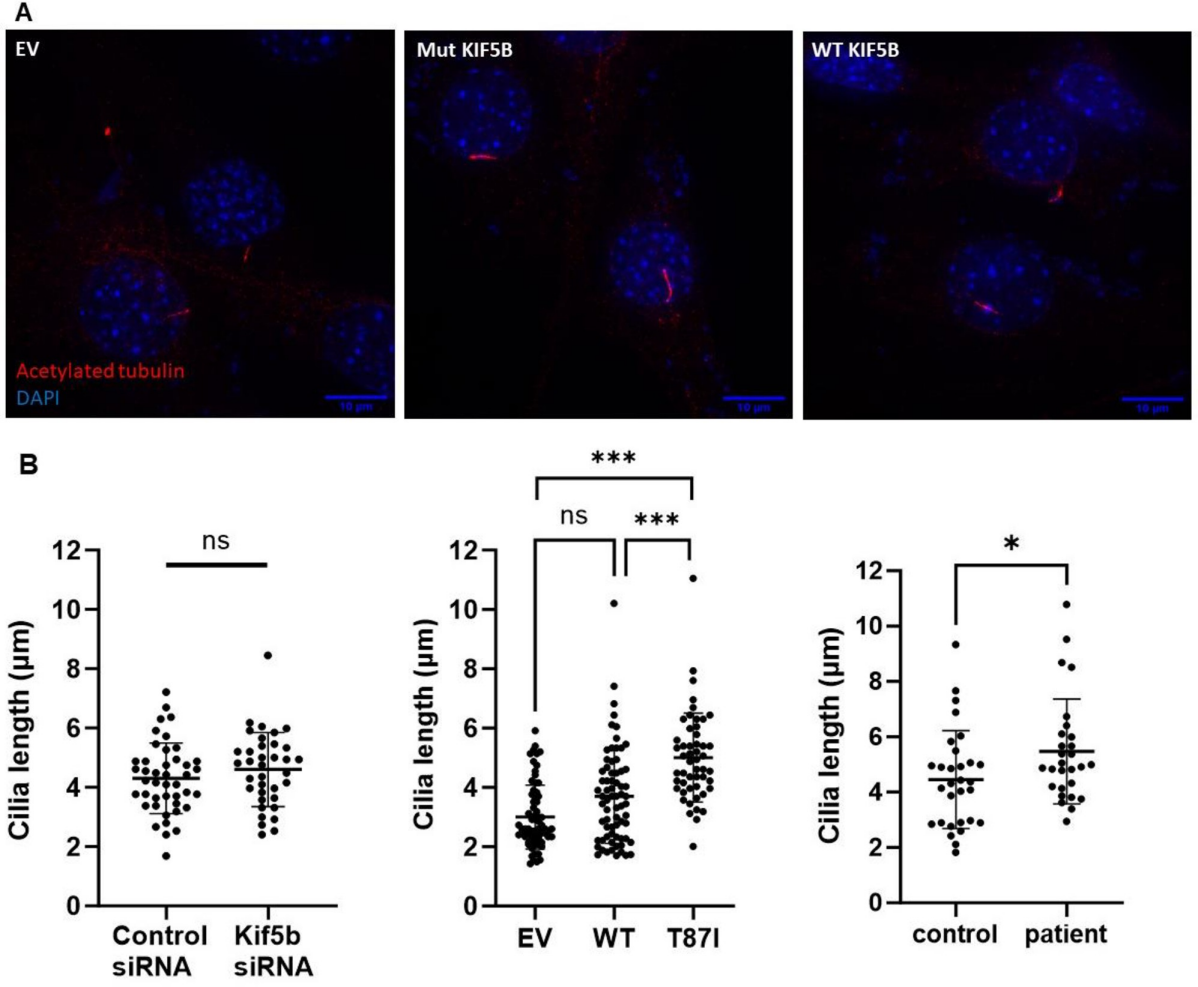
The *KIF5B* p.Thr87Ile variant is associated with elongated cilia. (A) Representative image showing elongated cilia in NIH3T3 cells overexpressing *KIF5B*^T87I/+^ (Mut, middle), compared to empty vector (EV, left) or wild type *KIF5B* (WT, right). (B) Left: Cilia length was not altered by KIF5B depletion (control siRNA n = 43, *Kif5b* siRNA n = 36). Middle: In contrast, overexpression of the mutant *KIF5B*^T87I^ was associated with elongation of the cilia, as compared to cells transfected with the wild-type allele or with empty vector (EV n = 69, WT n = 70, Mut n = 51). Right: Cilia length was significantly increased in primary patient’s fibroblasts (control human fibroblasts n = 30, proband 1 fibroblasts n = 28). T-test or one-way ANOVA, ns: not significant, *p<0.01, ***p<0.0001.

KIF5B has been implicated in the biogenesis of primary cilia and specifically was shown to regulate cilia length via its interaction with the BBSome complex [33]. The primary cilia were imaged in NIH3T3 cells overexpressing the human *KIF5B* p.Thr87Ile allele. Immunofluorescence labeling for acetylated tubulin demonstrated elongated primary cilia in cells expressing mutant *KIF5B* but not in cells overexpressing the wild type allele or in *Kif5b*-depleted cells (**Figs 5A, 5B** and **S4**). Similarly, the primary cilia were elongated in fibroblasts from proband 1 (**Fig 5B**).

### mTOR signaling is downregulated in *KIF5B* mutant cells

**Fig 6:**
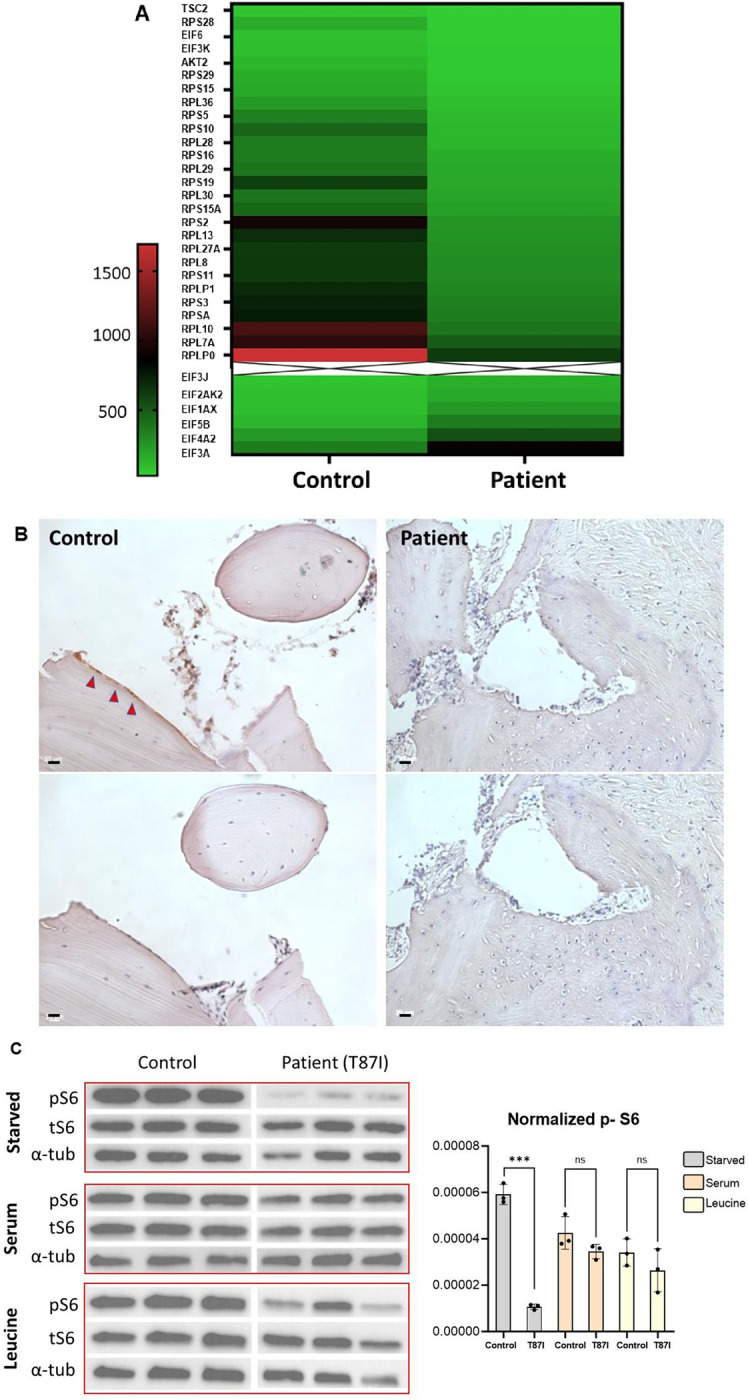
The *KIF5B* p.Thr87Ile variant is associated with reduced mTOR signaling. (A) Differential expression of curated gene list representing mTOR pathway, based on RNA sequencing analysis from proband 1 fibroblasts compared to UDN control group (top: transcripts that are downregulated in patient, bottom: transcripts that are upregulated in patient). (B) Bone biopsy from proband 1 (Patient) compared to control. Periosteal surface stained positive for phospho-S6 in control bone (red arrowheads), but not in the patient’s bone, which has immature and fibrotic bone matrix with hypercellularity that is typical of OI (Upper panel: Phospho-S6, bottom panel: 2^nd^ antibody only, x10 scale bar = 20μm). (C) Left: Western blot showing reduced phosphorylated S6 ribosomal protein (pS6) in serum-starved patient fibroblasts (top panel) compared to control cells. Results normalized to total S6 (tS6) and tubulin (α-tub). Refeeding with serum (middle panel), or leucine supplementation (bottom panel) reverses the phenotype in patient cells. Right: Quantification of immunoblot intensity using ImageJ (One-way ANOVA, n = 3 technical repeats, ns: not significant, ***p<0.0001).

Disruption of the Golgi-primary cilia axis can affect downstream intracellular signaling events [34–36]. To identify dysregulated signaling pathways, we performed differential gene expression analysis via mRNA sequencing in proband 1 fibroblasts as compared to Undiagnosed Diseases Network (UDN) control fibroblast transcriptome data (n = 131) [37]. The analysis revealed reduced expression of multiple genes encoding ribosomal proteins. Ingenuity Pathway Analysis (IPA) of the gene expression profile suggested downregulation of mTOR signaling upstream of these transcriptional alterations (**Fig 6A**). Immunohistochemistry staining for phosphorylated ribosomal protein S6 confirmed a decrease in mTOR signaling *in situ*, in proband 1 bone biopsy (**Fig 6B**). Western blot in proband 1 fibroblasts showed reduced phosphorylation of ribosomal protein S6, as well as decreased phosphorylation of AKT and mTOR proteins (**S5 Fig**). To potentially rescue this defect with a clinically translationally relevant intervention, we tested whether leucine amino acid supplementation could rescue this mTOR signaling defect. The essential amino acid leucine has been implicated in the regulation of mTOR signaling via mTORC1 activation [38–40]. As predicted, ribosomal protein S6 phosphorylation was restored after incubation with serum-containing media or by leucine supplementation (**Fig 6C**).

## Discussion

Kinesins are molecular motors that transport mRNA, proteins, and subcellular organelles within cells by moving the cargo along oriented microtubules [1–3]. *KIF5B*, encoding the ubiquitously expressed kinesin-1 heavy chain subunit, is essential for cell function and tissue development [10–14] and has been implicated in human disease [23,24]. We report here pathogenic variants in *KIF5B* in individuals with osteogenesis imperfecta. The variants that reside within the kinesin motor domain are predicted to interrupt nucleotide binding. Functional assessment of the recurrent p.Thr87Ile variant modeled in *C*. *elegans unc-116(T90I)* showed that the variant is damaging and disrupts post-embryonic development, body morphology, locomotion and kinesin motor activity via a dominant-negative mechanism. Studies in cells showed disrupted organization of the Golgi complex and altered cilia length, consistent with previous reports in *KIF5B*-mutated animal and cell culture models [12,24,33,41]. Importantly, downstream effect on the mTOR pathway was demonstrated in patient cells and bone tissue, highlighting a potential signaling defect that could be targeted for future therapy.

Kinesin-1 is an ATP-dependent motor protein [8], and the nucleotide-binding P-loop and switch loop I/II subdomains are critical to its activity. *In vitro* studies of key amino acid substitutions within the kinesin motor domain, including at Thr87, destabilized the nucleotide-binding pocket and favored an apo-kinesin (nucleotide-free) conformation that is strongly bound to microtubules [42–45]. The variants at Thr87, Gly90 and Thr195 are predicted to result in a catalytically inactive KIF5B protein due to destabilization of the active (ATP-bound) form (**Fig 1E**). In this scenario, heterozygous variants in the patients will likely have a dominant negative effect through dimerization of the catalytic-dead KIF5B with the wild type KIF5B. Of note, pathogenic variants affecting the homologous kinesin *KIF1A* have been proposed to act in a similar manner in autosomal dominant *KIF1A*-associated neurological disorder [46]. Consistent with this hypothesis, the *unc-116(Thr90Ile)* heterozygotes in *C*. *elegans* displayed dominant motility and body morphology phenotypes that were not observed in *unc-116* deletion heterozygotes. Moreover, these phenotypes were suppressed by expression of additional copies of the wild type allele supporting a dominant negative mechanism (**Fig 2**). Similarly, the altered ciliary length in cells expressing mutant p.Thr87Ile *KIF5B* was not observed in *Kif5b*-depleted cells, nor in cells overexpressing the wild type *KIF5B* (**Fig 5**). Clustering of the *KIF5B* variants p.Thr87Ile, p.Gly90Ala and p.Thr195Lys (reported here), as well as p.Lys91Arg and p.Gly234Val [23] within the kinesin motor domain suggests a genotype-phenotype correlation, compared to previously reported variants residing outside this domain that are not associated with skeletal findings [24] (**Fig 1D**).

Kinesins function in vesicle trafficking, including anterograde transport from the ER to the Golgi complex, trafficking within the Golgi cisternae, and transport between the Golgi, plasma membrane and other intracellular organelles [1,5,7,41,47]. Additionally, mouse *Kif5b* loss of function is known to disrupt mitochondrial transport [10,12]. Direct assessment of kinesin motor activity in *C*. *elegans unc-116(Thr90Ile)* heterozygotes showed reduced mitochondrial trafficking along microtubules in ALM neurites (**Fig 3**) providing support for the deleterious nature of the human p.Thr87Ile variant on kinesin function. Altered organization of the Golgi stacks was previously described in mouse *Kif5b*-deficient myogenic and chondrogenic cells [12,13], and in fibroblasts derived from individuals with *KIF5B*-related disorder [24]. In proband 1 fibroblasts, electron microscopy and immunofluorescence imaging demonstrated altered ER morphology, multiple vacuoles, and abnormal distribution of the Golgi complex (**Fig 4**). This pattern correlates with disruption of intracellular trafficking. Vesicular trafficking defects have been implicated in low bone mass phenotypes and bone fragility that are at least partly attributed to failure in collagen secretion [48–50]. Interestingly, impaired secretion of type II collagen has been demonstrated in *Kif5b*-deficient chondrocytes [14,51]. In proband 1 fibroblasts, type I collagen electrophoresis did not suggest a qualitative or quantitative collagen defect (**S1 Fig**). More research is needed to determine whether impaired collagen secretion is contributing to the phenotype in *KIF5B*-related osteogenesis imperfecta.

We also studied the effect of mutant KIF5B on cilia biogenesis. Overexpression of the wild type *KIF5B*, or its depletion by siRNA, did not significantly affect the cilia length. In contrast, expression of the mutant KIF5B^T87I^ was associated with elongated cilia, as has been observed in the proband 1 cells (**Fig 5**). This can be potentially explained by the direct effect of KIF5B on ciliogenesis via its interaction with CCDC28B at the ciliary basal body [33]. Alternatively, since primary cilia assembly and function depend on adequate protein transport through the Golgi, dysregulated cilia length may be secondary to the disruption of the secretory pathway in KIF5B^T87I^ mutant cells [52,53].

The secretory pathway initiates and propagates signal transduction, including the mitogen-activated protein kinase (MAPK) and mammalian target of rapamycin (mTOR) signaling pathways, via proteins residing in the ER and Golgi membranes [54]. In doing so, it not only regulates processes like cell proliferation and differentiation, but also modulates its own organization and function [54,55]. Our data demonstrated reduced mTOR signaling in patient fibroblasts and bone tissue (**Fig 6**). Santos-Ledo *et al*. had previously reported decreased mTOR phosphorylation and reduced phosphorylated ribosomal protein S6 in chondrocytes from *kif5b*-deficient zebrafish [14]. *KIF5B* plays an important role in lysosome positioning and in maintaining lysosome homeostasis, and KIF5B-depleted cells exhibit dysregulated mTOR signaling due to altered activation of the lysosome-associated mTORC1/mTORC2 complexes [56–59]. The Golgi apparatus also serves as a functional hub for mTOR proteins, and loss of Golgi integrity resulted in altered mTOR activity and induction of autophagy [60]. Thus, reduced mTOR signaling in KIF5B patients may be secondary to the disruption of organelle distribution and function in the setting of an intracellular trafficking defect. The dysregulation of mTOR signaling may also be related to altered primary cilia, or conversely may explain the abnormal ciliogenesis in cells expressing mutant KIF5B [61]. We have shown that loss of phosphorylated S6 in proband 1 cells can be partially rescued by supplementation with leucine, a potent stimulator of mTOR signaling (**Fig 6**). Leucine supplementation has been previously proposed as a therapeutic approach in other genetic disorders, including Roberts syndrome and Diamond-Blackfan anemia, that are also characterized by dysregulation of mTOR signaling, protein translation and autophagy [62–64]. Thus, our findings may have future therapeutic implications for patients with *KIF5B*-related disorder.

Pathogenic variants in *KIF5B* were previously reported in individuals with kyphomelic dysplasia, a skeletal dysplasia characterized by short stature, narrow thorax, bowing of the long bones and dysmorphic craniofacial features [23]. Probands 1–4 reported here were clinically diagnosed with osteogenesis imperfecta based on their history of osteopenia, multiple fractures, and bone deformities. The phenotypic overlap between reported patients, including short stature, bowed long bones, small chest, osteoporosis, fractures, and extra-skeletal manifestations (such as ptosis and hearing loss), suggests that these individuals may in fact be part of a clinical spectrum (Table 1 and [23]). Identification of additional individuals with *KIF5B* variants will allow for better characterization of the phenotype in *KIF5B*-related disorder.

One limitation of this study is the inability to model the skeletal dysplasia *in vivo*. We were unsuccessful in generating a *Kif5b*^*T87I*^ knock-in mouse model, likely due to early embryonic lethality, as has been reported in the *Kif5b* knockout mice [10]. The specific role of *KIF5B* in bone is not well-understood. An intracellular trafficking defect could potentially lead to failure in collagen secretion, and may be associated with ER stress, Golgi dysfunction, autophagy, and altered signaling, ultimately affecting osteo-chondroprogenitor cells’ differentiation or extracellular matrix composition. KIF5B deficiency was associated with a defect in cartilage development and skeletal mineralization in zebrafish and in the conditional Col2a1-Cre mouse model [13,14]. We had shown down regulation of the mTOR signaling pathway, which plays a critical role in bone development. Notably, reduced mTOR signaling has been previously implicated in mouse models of osteogenesis imperfecta [65–67]. Together, this provides a mechanistic rationale for the abnormal skeletal phenotype in the patients.

In summary, we have identified heterozygous variants in *KIF5B* as a novel cause of osteogenesis imperfecta with a dominant negative mechanism due to an intracellular trafficking defect and downregulation of mTOR signaling pathway. This study emphasizes the critical role of KIF5B and intracellular trafficking in maintaining skeletal homeostasis.

## Methods

### Ethics statement

For human studies, written informed consent was obtained from participants, or from their parent/legal guardian, as part of the Undiagnosed Diseases Network (UDN) for probands 1 and 2, according to the institutional review board of the National Human Genome Research Institute (15HG0130). Proband 1 was also enrolled to a research protocol for skeletal dysplasias that was approved by the Baylor College of Medicine Institutional Review Board (H-25722). Probands 3 and 4 were ascertained by diagnostic testing procedures, and their legal guardians gave clinical informed consent for testing. This was obtained using standard forms at local site by the responsible referring physicians.

### Exome sequencing and identification of *KIF5B* variants

*Proband 1* had research trio exome sequencing performed at the Baylor College of Medicine Human Genome Sequencing Center (BCM-HGSC). Briefly, library was constructed as described in the BCM-HGSC protocol and hybridized to the HGSC VCRome 2.1 design (42Mb NimbleGen, Cat. No. 06266380001) [68]. Sequencing was performed in paired-end mode using the Illumina HiSeq 2000 platform. With a sequencing yield of 9.1 Gb, the samples achieved an average coverage of 106x and 93.4% of the targeted exome bases were covered to a depth of 20X or greater. Illumina sequence analysis was performed using the HGSC Mercury analysis pipeline [69,70] which moves data through various analysis tools from the initial sequence generation on the instrument to annotated variant calls. In parallel to the exome workflow an Illumina Infinium Exome-24 v1.1 array was generated for a final quality assessment. This included orthogonal confirmation of sample identity and purity using the Error Rate In Sequencing (ERIS) pipeline developed at the HGSC. A successfully sequenced sample met quality control metrics of ERIS SNP array concordance (>90%) and ERIS average contamination rate (<5%). Variants were reviewed using Codified Genomics software (https://www.codifiedgenomics.com). We prioritized variants that were de novo, biallelic, and X-linked with a minor allele frequency of <0.01% within gnomAD (https://gnomad.broadinstitute.org/) [25] and our internal database. *Proband 2* had research whole genome sequencing performed at Baylor Genetics. In brief, genomic DNA was fragmented and indexed to prepare individual library, followed the PCR free 550-bp insert size protocol by the KAPA Hyper Prep kit. The library was subjected to sequence analysis on Illumina NovaSeq 6000 platform for 150 bp paired end reads with average sequenced coverage over the genome > 40X. Data analysis and interpretation were performed by the Baylor Genetics analytics pipeline. *Probands 3 and 4* underwent diagnostic trio exome sequencing in the Exeter Genomics Laboratory (Royal Devon University Healthcare NHS Foundation Trust, Exeter, UK). Libraries were prepared and targets captured using the Twist Exome kit (Twist Bioscience, South San Francisco, CA, USA) according to the manufacturer’s instructions. Sequencing was carried out on an Illumina NextSeq550 (Illumina, San Diego, CA, USA) using 150-bp paired end reads. All libraries met minimum quality requirements of mean depth 80X or greater and >95% of targeted bases covered to a depth of 20X or greater. Variants were called in human genome build GRCh37 and annotated using Alamut Batch software (Interactive Software, Rouen, France).

### RNA sequencing

Messenger RNA from fibroblasts of proband 1 and UDN controls was extracted, quantified, and processed, prior to library preparation using the Illumina TruSeq kit (Illumina, SD) according to manufacturer’s instructions. Samples were subsequently multiplexed and subject to 150bp paired-end sequencing on the Illumina HiSeq2000 platform. Resulting raw bcl reads were converted to FASTQ prior to being processed using a pipeline adapted from the Genotype-Tissue Expression (GTEX) Consortium [71]. Briefly, reads were aligned to the GRCh37 human reference using Spliced Transcript Alignment to a Reference (STAR) [72], followed by gene-based quantification with RNA-Seq by Expectation Maximization (RSEM) [73] and alignment visualization in Integrative Genomics Viewer (IGV) [74]. For gene expression analysis, FPKM values (fragments per kilobase of transcript per million fragments mapped) in the proband RNA-seq data were compared to the mean FPKM value from pooled control RNA-seq data. The p value, mean and standard deviation were calculated based on the normal distribution of FPKM values among samples. Z score values were calculated as (observed FPKM-Mean)/standard deviation. The significantly differential expressed genes (fold change >2 and p value <0.001) were then uploaded into IPA (Qiagen) for upstream regulator prediction.

### Protein 3D structure analysis

Structural impact of variants was assessed in structures of KIF5B bound to either ADP or ATP (Protein Data Bank structures 1mkj and 3j8y, respectively). Variants were introduced by *in silico* mutagenesis using the FoldX modeling suite [75]. Structures were visualized and images generated using PyMOL (PyMOL Molecular Graphics System, Version 2.0, Schrödinger LLC; New York, NY, USA).

### Electron microscopy

Fibroblast cells were pelleted and fixed in 4% paraformaldehyde, washed, and dehydrated in a graded ethanol series from 30–100%. The samples were then washed in propylene oxide and embedded in Spurrs epoxy. Ultrathin sections were stained in uranyl acetate followed by Reynolds lead citrate and examined using a FEI Tecnai G2 TEM.

### Cell culture

All cells were cultured at 37°C and 5% CO_2_ in a humid environment. Primary human fibroblasts, or NIH3T3 cells were cultured in growth medium (alpha MEM supplemented with 10% fetal bovine serum, 1% glutamine and 1% penicillin/streptomycin). NIH3T3 cells were transiently transfected with either wild type *KIF5B*, mutant *KIF5B*^T87I^ (GenScript GenEZ ORF clone in pcDNA3.1 flag-tag) or empty vector (pcDNA3.1) using Lipofectamine 2000 reagent (Thermofisher). To knockdown *Kif5b* in NIH3T3 cells, cells were transfected with ON-TARGETplus Mouse *Kif5b* siRNA SMARTPool (Horizon) using Lipofectamine RNAiMax (Thermofisher). Total cell lysates were analyzed by western blot and real-time PCR to quantify KIF5B level. For measurements of cilia length, cells were subject to overnight starvation in growth media containing 0.5% fetal bovine serum. For studying mTOR pathway, cells were starved in serum-free media for 6 hours prior to sample collection. For rescue experiments, L-Leucine (AthenaES, 5mM) or fetal bovine serum (10%) were added to the serum-free media for 30 minutes prior to sample collection.

### Type I collagen analysis

Primary human fibroblasts were cultured overnight in serum-free alpha MEM containing 50 uM ascorbic acid (Sigma) to stimulate collagen secretion. Media was harvested the next day, and protein was extracted by heat denaturation in SDS-PAGE sample buffer. Type I collagen α-chains were resolved on 6% SDS-PAGE gel, then processed by trypsin in-gel digestion [76]. Digested fractions were analyzed for hydroxylation of proline residues by Liquid Chromatography/Mass spectrometry (LC/MS, Thermo Scientific).

### Protein extraction and western blot

Protein was extracted from whole cell lysates in RIPA buffer. The protein concentration was measured using a BCA kit (ThermoFisher Scientific). For western blot analysis, 10–20 ug of total protein was loaded on 4–15% SDS-PAGE gradient gel (Bio-Rad) and transferred to PVDF membrane (Millipore Sigma). Membrane was blocked in 5% skim milk and incubated overnight at 4°C with primary antibody. The following primary antibodies were used: anti- DDDDK tag (Abcam #ab1257), anti- phospho-S6 ribosomal protein (Ser235/236) (Cell Signaling #2211), anti-S6 ribosomal protein (Cell Signaling #2217), anti-AKT (Cell Signaling #4691), anti-phospho AKT (Cell Signaling #9271), anti-mTOR (Cell Signaling #2983), anti-phospho mTOR (Cell Signaling #5536), and anti-alpha Tubulin (Sigma, T5168). Signal was captured via ChemiDoc imager (Bio Rad).

### RNA extraction and real-time qPCR

Total RNA was extracted from cell lysates in Trizol reagent (Ambion). The iScript cDNA synthesis kit (Bio-Rad) was used to synthesize cDNA. Real-time qPCR was performed on LightCycler instrument (Roche) using FastStart Essential DNA Master reagent (Roche) with *B2m* (beta-2 microglubulin) as internal control.

### Immunofluorescence microscopy

Cells were cultured in chamber slides, fixed in 4% formaldehyde, blocked with 5% serum in PBS/0.3%Triton X-100, and incubated overnight at 4°C with primary antibody. Primary antibodies used in this study were: anti-GM-130 (Cell Signaling #12480) and rabbit anti-acetyl-α-Tubulin (Cell Signaling #5335). Secondary antibody was donkey anti-rabbit IgG (H+L), Alexa Fluor 594 (Thermofisher #A-21207). Slides were mounted using Prolonged Gold Antifade mounting medium with DAPI (Thermofisher). Acquisition of images was done with the assistance of the Baylor College of Medicine Integrated Microscopy Core, using Cytivia DVLive epifluorescence deconvolution microscope. Images were captured on a sCMOS camera taking z-stacks with 0.2μm optical sections. Cilia length was measured using ImageJ.

### Golgi imaging in human primary fibroblasts

Quantitative imaging of Golgi structure and distribution was performed in human skin fibroblasts from proband 1 or control cells. Fibroblasts were cultured in chamber slides, and Golgi complex was labeled by CellLight Golgi-GFP BacMan 2.0 reagent per manufacturer’s protocol (ThermoFisher Scientific). Staining with Hoechst was used to label nuclei. Acquisition and analysis of images was done with the assistance of the Baylor College of Medicine Integrated Microscopy Core, using Vala IC200 high throughput microscope. Nuclear size and intensity, as well as number of Golgi structures, area, mean pixel intensity, and maximum length were measured. A measurement scale of 0 to 1 was used to identify the relative radial distribution of each Golgi. Golgi circularity was scored on a scale of 0 to 1, with 0 representing a Golgi that is polarized to one region, and a score of 1 representing a fully dispersed Golgi around the nucleus.

### Immunohistochemistry in bone biopsy

Bone samples from proband 1 and unaffected control were obtained when subjects were undergoing skeletal surgery for a medical indication. The fragments of bone removed during surgery, which otherwise would have been discarded, were processed for histology. In brief, bone samples were fixed in 4% paraformaldehyde for 48 hours and decalcified in 10% EDTA (Millipore Sigma) for 14 days at 4°C. Paraffin embedded tissue was sectioned, deparaffinized, and incubated at 37°C with 0.05% trypsin for antigen retrieval (Abcam ab970), followed by 3% hydrogen peroxide treatment. After blocking with 5% normal goat serum, sections were incubated with primary antibody anti-phospho-S6 ribosomal protein (Ser235/236) (Cell Signaling #2211) overnight at 4°C. Anti-rabbit secondary antibody and DAB staining (Vector Laboratories, Vectastain Elite ABC-HRP Kit #PK-6101) were applied per manufacturer’s instructions. Slides were stained with Hematoxylin and mounted using Cytoseal XYL xylene-based mounting medium (Thermo Scientific). Images were taken with a light microscope (Axioplan 2, Zeiss).

### Modeling in *C*. *elegans*

#### *C*. *elegans* culture and strain information

*C*. *elegans* strains were cultured on standard nematode growth media (NGM) seeded with *Escherichia coli* OP50 and grown at 20°C. Strains used in this paper include *jsIs609 [mec7p*::*mtGFP + lin-15(+)]* X and VC2010. *unc-116* variant edits, control edits, null deletion and single copy integration strains generated in this study are described below. A full list of strains used in this study is in **S1 Table**. Some strains were provided by the CGC, which is funded by NIH Office of Research Infrastructure Programs (P40 OD010440).

#### *C*. *elegans* genome editing

*unc-116* edits were generated as previously described [77]. In brief, VC2010 adults were injected with Cas9 (IDT 1081060), tracrRNA (IDT 1072533), crRNA targeting sequence GCATATGGACAAACATCTTC (IDT CRISPR-Cas9 sgRNA), single-stranded DNA oligonucleotide repair template (IDT), and *dpy-10* crRNA and repair template (the co-conversion marker) [78,79]. The variant repair template includes the proband edit and synonymous changes, which destroy Cas9 re-targeting and create a TspRI restriction site for genotyping. The control repair template with the synonymous changes, but not the proband variant, was used to generate control edit lines to verify that the synonymous changes do not contribute to the observed phenotype in the variant edit animals. Three T90I variant edit lines and two T90T control edit lines were initially generated. T90I#1, T90I#2 and T90T#1 were Sanger sequenced verified and outcrossed twice to remove any potential background mutations. Null deletion allele *unc-116(gk5722udn86)* is derived from *unc-116(gk5722[loxP + myo-2p*::*GFP*::*unc-54 3’ UTR + rps-27p*::*neoR*::*unc-54 3’ UTR + loxP])*. Selection cassette (*myo-2p*::*GFP*::*unc-54 3’ UTR + rps-27p*::*neoR*::*unc-54 3’ UTR*) in *unc-116(gk5722)* was removed by injecting a plasmid pDD104 expressing Cre recombinase (a gift from Bob Goldstein, Addgene plasmid # 47551) as described previously [80]. The single-copy *unc-116(+)* transgene was integrated at the Mos1 transposon insertion site *ttTi5605* on chromosome II (II: 0.77cM) using recombination-mediated cassette exchange (RMCE) [29,30]. The entire *unc-116* genomic locus was amplified from N2 genomic DNA using oligonucleotides 6932 and 6947, digested with MluI and SbfI and inserted into pLF3FShC. The resulting plasmid NM3933 was injected at 50 ng/μl into NM5340 *jsSi1579 [loxP rpl-28p FRT GFP*::*his-58 FRT3] II; unc-119(ed3) III; bqSi711 [mex-5pFLP sl2 mNeon Green] IV*. Three independent integrants were obtained as previously described [81]. After outcrossed twice with VC2010 to remove *unc-119(ed3) III* and *bqSi711 [mex-5pFLP sl2 mNeon Green] IV*, three single copy *unc-116(+)* transgene lines were generated.

#### The *unc-116* transgene RNA-seq analysis

To facilitate the comparison of mRNA level between the *unc-116* wild type and transgene locus in RNA-seq analysis, we have introduced four synonymous substitutions in exon 3 at the endogenous locus to generate *unc-116(udn42)*. We conducted the RNA-seq analysis in triplicates to compare the mRNA level/read counts between the *unc-116* wild type locus (chromosome III) and single copy transgene locus on chromosome II, using the strain UDN100221. The UDN100221 harbors the *unc-116* transgene allele *jsSi1579 jsSi1606* and the edited allele *und42* at the endogenous locus [e.g., *unc-116(udn42)*]. In brief, 1-day old young adults were collected to isolate total RNA with Trizol reagent following the manufacturer’s instruction. Total RNA was further purified with DNase digestion followed by column extraction. The following Next Generation Sequencing (NGS) DNA library preparation and bioinformatic analysis were conducted by Genome Technology Access Center: ~10 μg of total RNA each was used to enrich mRNA before DNA library preparation, using Oligo-dT beads (mRNA Direct kit, Life Technologies). mRNA was first fragmented in reverse transcriptase buffer by incubation at 94°C for 8 minutes. mRNA was then reverse transcribed to yield cDNA using the SuperScript III RT enzyme (Life Technologies) with random hexamers. A second strand reaction was performed to yield double-strand cDNA. cDNA was blunt ended, had an A base added to the 3’ ends, and then Illumina sequencing adapters were ligated to the ends. Ligated fragments were then PCR amplified for 12–15 cycles, using primers to integrate unique dual index tags for the different biological replicates. Fragments were sequenced on an Illumina NovaSeq-6000 using paired-end reads extending 150 bases. The resulting reads were then quasi-aligned and quantitated against the Ensembl WBcel235.90 transcriptome with copies of the wild type and transgenic *unc-116* using the expectation-maximization algorithm in Salmon [82] to generate allele-specific expression in transcripts per million (TPM). The results indicate that the mRNA level of the single copy *unc-116* transgene is not significantly different from the endogenous locus, and thus can be considered as supplying an equivalent gene dose as the wild type *unc-116* gene.

#### *C*. *elegans* body length and swimming analysis

Body length and swimming speed were measured using the WormLab 2019.1.1 (MBF Bioscience) [81]. Briefly, about 30 late L4 stage worms per strain were transferred onto seeded NGM plates 24 hours before the assay. Meanwhile, 100 mm NGM plates were seeded with 50 μL 10x concentrated OP50, spreading onto the plate surface evenly, and allowed to dry for 1 day. On the day of assay, 10 animals were transferred to one newly made assay plate, recovering for 20 minutes, and recorded for 2 minutes of worm crawling. After that, 1mL PBS was added into the plate, waiting for 30 seconds for worms to adapt to swimming, and then 2 minutes of worm swimming video was captured. Three plates per strain were recorded per trial and three independent trials were performed for each strain, totaling about 90 animals per strain. Videos were analyzed using WormLab software. When multiple tracks were generated by one worm, tracks were manually joined when possible, otherwise the longer duration track was used. Any worms that recorded less than 15 seconds were censored from analysis.

#### Generation of variant heterozygotes

To obtain heterozygotes, L4 stage animals were transferred to *fem-1* RNAi plates to generate females. Adult females, identified by presence of stacked oocytes but not embryos, were selected to cross with males of appropriate genotype. Therefore, the yielding offspring were all cross progeny. Specifically, for *unc-116*/*qC1*::GFP, females were crossed with VC2010 males and heterozygote progeny without pharyngeal GFP were selected for phenotyping.

#### Image acquisition and analysis

For imaging UNC-116 mediated transport of mitochondria along axonal microtubules, L4 worms with GFP tagged mitochondria in mechanosensory neurons were immobilized with 10 mM levamisole, mounted on 2.5% agarose pads, and sealed with Vaseline. 5 minutes time-lapse images (1fps) were acquired using 63x objective on a spinning disk confocal microscope (PerkinElmer). Time-lapse images were converted into 2D kymographs by KymoResliceWide plugin in ImageJ. Images were aligned with StackReg Plugin in ImageJ where worm movement was detected during imaging. 100 μm long neurite regions, starting from ALM soma, were used for quantification of mitochondria moving particles. Particles with more than 3 μm displacement were counted as moving particles. Anterograde moving particles were defined by particles moving away from the cell body. Net run length was calculated as the distance between the starting position and the stop position. For combined segmental velocity, each trajectory was manually tracked using segmented line tool and processed automatedly by KymoAnalyzer [32] in ImageJ. For images in **Fig 2**, worms were imaged with Leica M165 FC Fluorescent Stereo Microscope. For confocal images in **Fig 3**, “Straighten” function of ImageJ was used to straighten curved ALM neurites.

#### Statistical analysis

Statistical analysis was performed using GraphPad Prism (Version 8.0.2). Student’s t-test or Mann-Whitney test were used for comparing differences between two groups, while one-way ANOVA or Kruskal-Wallis test followed by Dunn’s multiple comparison was performed for comparing three or more groups. For *C*. *elegans* modeling: Data were shown as median value, except Figs 2B and S**3**A in which mean and SD were shown. For cell studies: Data were shown as mean value and SD. Statistical significance was determined as p value that is equal to, or less than 0.01.

## Supporting information

S1 Movie Control *C*. *elegans* (WT, VC2010) swimming. (AVI)

S2 Movie *unc-116(T90I)* variant (Het) swimming. (AVI)

S3 Movie *unc-116(T90T)* control edit mitochondrial transport. (AVI)

S4 Movie *unc-116(T90I)* variant (Het) mitochondrial transport. (AVI)

S1 Data Supplemental data file, RNA sequencing data. (XLSX)

S2 Data Supplemental data file, numerical data (cell studies). (XLSX)

S3 Data Supplemental data file, numerical data (*C*.*elegans* studies). (XLSX)

S1 Fig Collagen post-translational modification is not altered in Proband 1. Type I collagen isolated from patient fibroblasts showed no difference in electrophoretic migration pattern between patient and control. Mass spectrometry analysis showed no difference in hydroxylation pattern of 3-Hydroxyproline at P986 between patient and control.
(PDF)

S2 Fig *KIF5B* P-loop nucleotide binding domain is highly conserved between species. Multiple sequence alignment of the N-terminal part of the motor domain of *KIF5B* and orthologs in other organisms. The highly conserved P-loop region of the ATP-binding domain is highlighted with a green box. The KIF5B p.Thr87Ile variant in proband 1 (red arrowhead) and corresponding *C*. *elegans* (worm) *unc-116* Thr90 residue (blue arrowhead) are shown.
(PDF)

S3 Fig Single copy transgene of *unc-116(+)* is functional. (A) RNA sequencing transcripts per million (TPM) of endogenous *unc-116* and *unc-116* transgene on chromosome *II*. Rescue of body length (B) and thrashing speed (C) defects were demonstrated upon introduction of two additional copies of *unc-116(+)* transgene into *unc-116 (del)*. The difference in length between wild type and *unc-116(del)* with 2 extra copies of *unc-116(+)* is less than 2% and while statistically different is not considered biologically significant. Student t-test was used for comparison; ns: not significant, **p<0.001.
(PDF)

S4 Fig *KIF5B* overexpression and knockdown in NIH3T3 cells. (A) western blot analysis (left) and real time RT-PCR (right) showing overexpression of Flag-tagged wild type and mutant *KIF5B* in transiently transfected NIH3T3 cells (EV- empty vector, WT- wild type KIF5B, MUT-KIF5B^T87I^). (B) Real time RT-PCR showing efficient knock-down of *Kif5b* in NIH3T3 cells using siRNA. ns: not significant, *p<0.01, **p<0.001, ***p<0.0001.
(PDF)

S5 Fig Reduced mTOR signaling in patient fibroblasts. Western blot showing reduced phosphorylated AKT (top panel) and mTOR (bottom panel) in serum-starved patient fibroblasts.
(PDF)

S1 Table List of *C*. *elegans* strains used in this study. (PDF)

S2 Table *unc-116* genome editing reagents. (PDF)

S1 Acknowledgement List of members of the UDN. (PDF)
