# Supplementary material for: Dominant negative variants in KIF5B cause osteogenesis imperfecta via down regulation of mTOR signaling
Source: PLoS Genet. 2023 Nov 7;19(11):e1011005. doi: 10.1371/journal.pgen.1011005 (PMC10656020; doi:10.1371/journal.pgen.1011005)
Supplement: S1 Fig — Type I collagen isolated from patient fibroblasts showed no difference in electrophoretic migration pattern between patient and control. Mass spectrometry analysis showed no difference in hydroxylation pattern of 3-Hydroxyproline at P986 between patient and control. (PDF) [file pgen.1011005.s008.pdf]

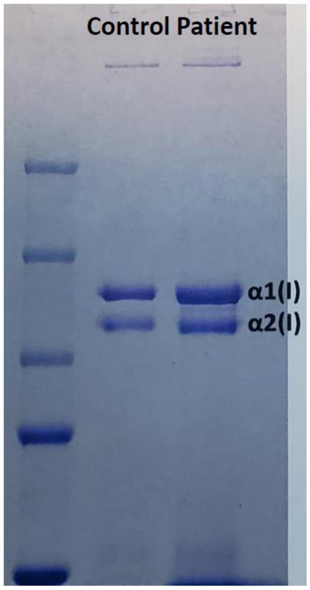

	P986-3Hyp
**Control**	98%
**Patient**	98%

S1 Fig. Collagen post-translational modification is not altered in Proband 1. Type I collagen isolated from patient fibroblasts showed no difference in electrophoretic migration pattern between patient and control. Mass spectrometry analysis showed no difference in hydroxylation pattern of 3-Hydroxyproline at P986 between patient and control.
