# Supplementary material for: Dominant negative variants in KIF5B cause osteogenesis imperfecta via down regulation of mTOR signaling
Source: PLoS Genet. 2023 Nov 7;19(11):e1011005. doi: 10.1371/journal.pgen.1011005 (PMC10656020; doi:10.1371/journal.pgen.1011005)
Supplement: S2 Fig — Multiple sequence alignment of the N-terminal part of the motor domain of KIF5B and orthologs in other organisms. The highly conserved P-loop region of the ATP-binding domain is highlighted with a green box. The KIF5B p.Thr87Ile variant in proband 1 (red arrowhead) and corresponding C. elegans (worm) unc-116 Thr90 residue (blue arrowhead) are shown. (PDF) [file pgen.1011005.s009.pdf]

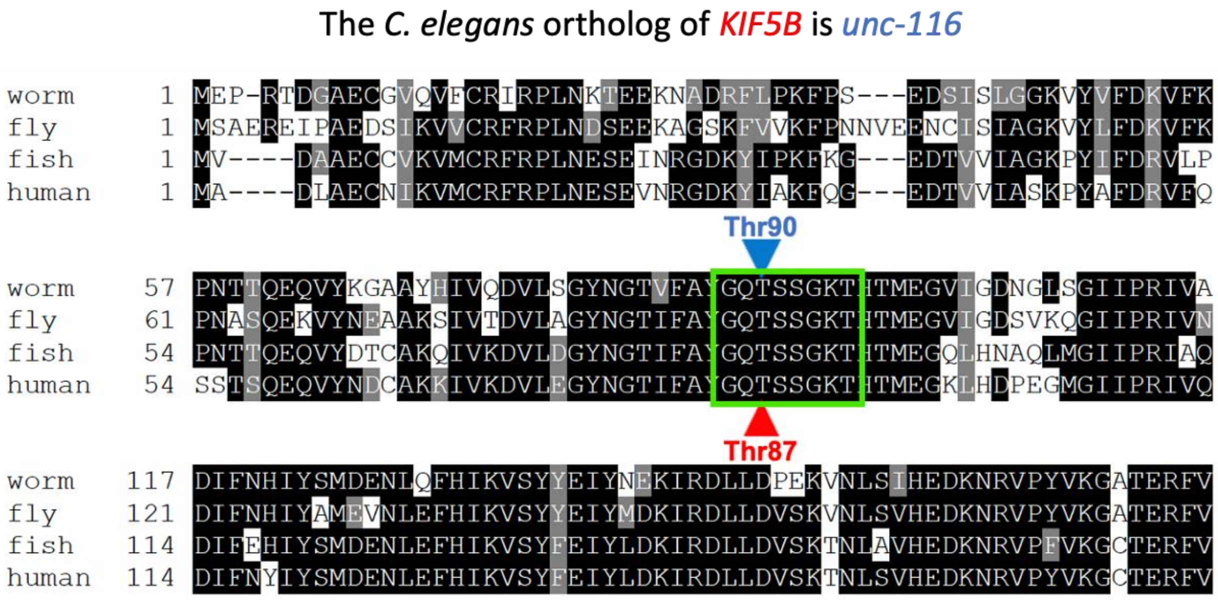

S2 Fig. *KIF5B* P-loop nucleotide binding domain is highly conserved between species. Multiple sequence alignment of the N-terminal part of the motor domain of *KIF5B* and orthologs in other organisms. The highly conserved P-loop region of the ATP-binding domain is highlighted with a green box. The KIF5B p.Thr87Ile variant in proband 1 (red arrowhead) and corresponding *C. elegans* (worm) *unc-116* Thr90 residue (blue arrowhead) are shown.
