# Supplementary material for: Dominant negative variants in KIF5B cause osteogenesis imperfecta via down regulation of mTOR signaling
Source: PLoS Genet. 2023 Nov 7;19(11):e1011005. doi: 10.1371/journal.pgen.1011005 (PMC10656020; doi:10.1371/journal.pgen.1011005)
Supplement: S3 Fig — (A) RNA sequencing transcripts per million (TPM) of endogenous unc-116 and unc-116 transgene on chromosome II. Rescue of body length (B) and thrashing speed (C) defects were demonstrated upon introduction of two additional copies of unc-116(+) transgene into unc-116 (del). The difference in length between wild type and unc-116(del) with 2 extra copies of unc-116(+) is less than 2% and while statistically different is not considered biologically significant. Student t-test was used for comparison; ns: not significant, **p<0.001. (PDF) [file pgen.1011005.s010.pdf]

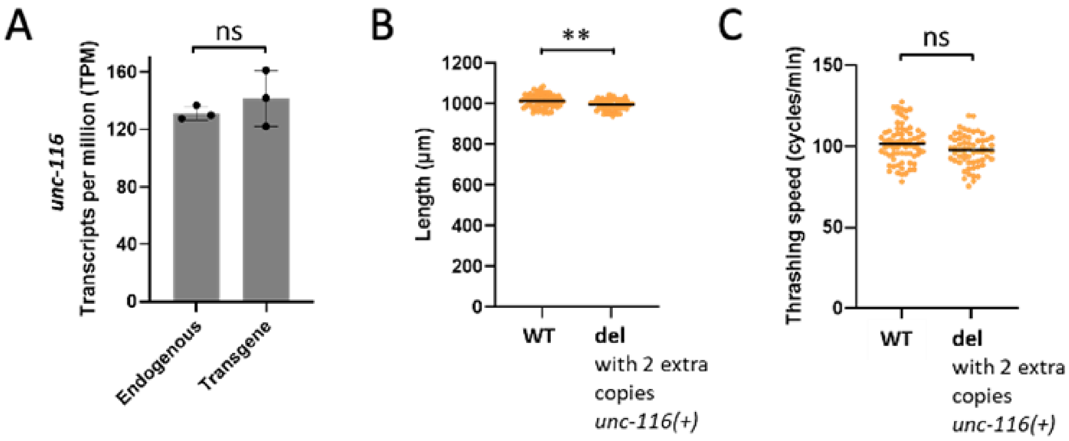

S3 Fig. Single copy transgene of *unc-116(+)* is functional. (A) RNA sequencing transcripts per million (TPM) of endogenous *unc-116* and *unc-116* transgene on chromosome *II*. Rescue of body length (B) and thrashing speed (C) defects were demonstrated upon introduction of two additional copies of *unc-116(+)* transgene into *unc-116 (del)*. The difference in length between wild type and *unc-116(del)* with 2 extra copies of *unc-116(+)* is less than 2% and while statistically different is not considered biologically significant. Student t-test was used for comparison; ns: not significant, **p<0.001.
