# Supplementary material for: Dominant negative variants in KIF5B cause osteogenesis imperfecta via down regulation of mTOR signaling
Source: PLoS Genet. 2023 Nov 7;19(11):e1011005. doi: 10.1371/journal.pgen.1011005 (PMC10656020; doi:10.1371/journal.pgen.1011005)
Supplement: S4 Fig — (A) western blot analysis (left) and real time RT-PCR (right) showing overexpression of Flag-tagged wild type and mutant KIF5B in transiently transfected NIH3T3 cells (EV- empty vector, WT- wild type KIF5B, MUT-KIF5BT87I). (B) Real time RT-PCR showing efficient knock-down of Kif5b in NIH3T3 cells using siRNA. ns: not significant, *p<0.01, **p<0.001, ***p<0.0001. (PDF) [file pgen.1011005.s011.pdf]

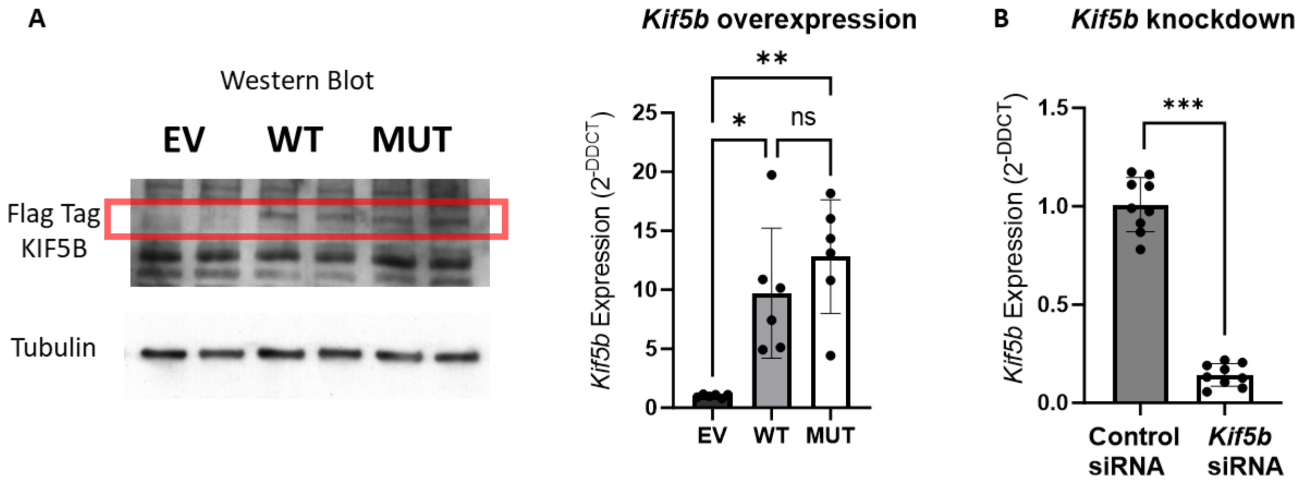

S4_Fig. *KIF5B* overexpression and knockdown in NIH3T3 cells. (A) western blot analysis (left) and real time RT-PCR (right) showing overexpression of Flag-tagged wild type and mutant *KIF5B* in transiently transfected NIH3T3 cells (EV- empty vector, WT- wild type KIF5B, MUT- $KIF5B^{T87I}$). (B) Real time RT-PCR showing efficient knock-down of *Kif5b* in NIH3T3 cells using siRNA. ns: not significant, *p<0.01, **p<0.001, ***p<0.0001.
