# Supplementary material for: Dominant negative variants in KIF5B cause osteogenesis imperfecta via down regulation of mTOR signaling
Source: PLoS Genet. 2023 Nov 7;19(11):e1011005. doi: 10.1371/journal.pgen.1011005 (PMC10656020; doi:10.1371/journal.pgen.1011005)
Supplement: S5 Fig — Western blot showing reduced phosphorylated AKT (top panel) and mTOR (bottom panel) in serum-starved patient fibroblasts. (PDF) [file pgen.1011005.s012.pdf]

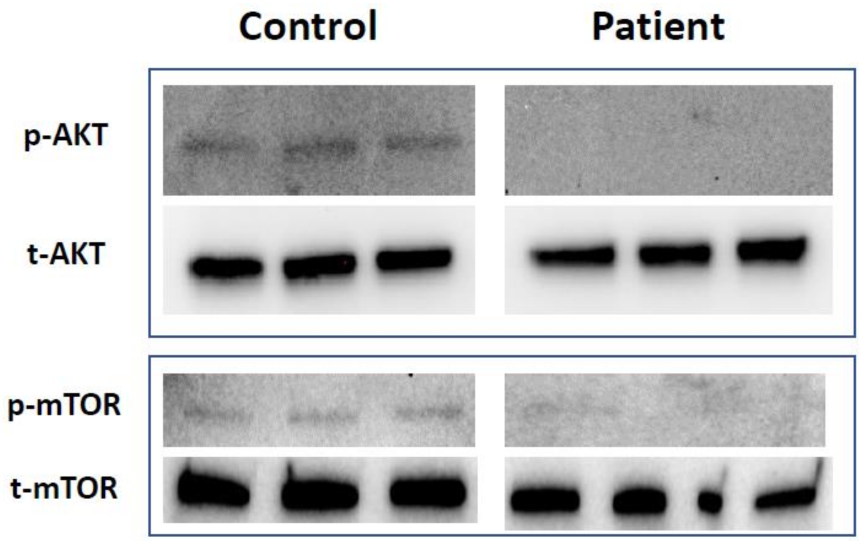

S5 Fig. Reduced mTOR signaling in patient fibroblasts. Western blot showing reduced phosphorylated AKT (top panel) and mTOR (bottom panel) in serum-starved patient fibroblasts.
