# Supplementary material for: Dominant negative variants in KIF5B cause osteogenesis imperfecta via down regulation of mTOR signaling
Source: PLoS Genet. 2023 Nov 7;19(11):e1011005. doi: 10.1371/journal.pgen.1011005 (PMC10656020; doi:10.1371/journal.pgen.1011005)
Supplement: S1 Table — (PDF) [file pgen.1011005.s013.pdf]

S1 Table. List of *C. elegans* strains used in this study

Strain Name	Genotype	Description
VC2010	Wild type	Wild type
UDN100185	*unc-116(udn42)/qC1 [dpy-19(e1259) glp-1(q339)] nIs189 III*	Control-edited allele T90T #1.<br>*nIs189 [myo-2p::GFP]* integrated in or near *qC1*.
UDN100082	*unc-116(udn44)/qC1 [dpy-19(e1259) glp-1(q339)] nIs189 III*	Variant-edited allele T90I #1.
UDN100083	*unc-116(udn45)/qC1 [dpy-19(e1259) glp-1(q339)] nIs189 III*	Variant-edited allele T90I #2.
UDN100169	*unc-116(gk5722udn86)/qC1 [dpy-19(e1259) glp-1(q339)] nIs189 III*	Null deletion.
NM5340	*jsSi1579 [loxP rpl-28p FRT GFP::his-58 FRT3] II; unc-119(ed3) III; bqSi711 [mex-5pFLP sl2 mNeon Green] IV*	RMCE landing site [1]
UDN100201	*jsSi1579 jsSi1606 [unc-116(+)] II*	*unc-116(+)* single copy insertion.
UDN100221	*jsSi1579 jsSi1606 [unc-116(+)] II; unc-116(udn42) III*	T90T with extra copy *unc-116*
UDN100222	*jsSi1579 jsSi1606 [unc-116(+)] II; unc-116(udn45) III*	T90I with extra copy *unc-116*
UDN100242	*jsSi1579 jsSi1606 [unc-116(+)] II; unc-116(gk5722udn86) III*	Null with extra copy *unc-116*
NM2271	*jsIs609 [mec7p::mtGFP + lin-15(+)] lin-15(n765) X*	GFP-tagged mitochondria expressed in TRNs [2]
UDN100238	*unc-116(udn42)/qC1 [dpy-19(e1259) glp-1(q339)] nIs189 III; jsIs609 [mec7p::mtGFP + lin-15(+)] lin-15(n765) X*	T90T with GFP-tagged mitochondria.
UDN100239	*unc-116(udn45)/qC1 [dpy-19(e1259) glp-1(q339)] nIs189 III; jsIs609 [mec7p::mtGFP + lin-15(+)] lin-15(n765) X*	T90I with GFP-tagged mitochondria.

References:

1. Nonet M. Additional Landing Sites for Recombination-Mediated Cassette Exchange in C. elegans. MicroPubl Biol. 2021;2021. Epub 2021/12/23. doi: 10.17912/micropub.biology.000503. PubMed PMID: 34934910; PubMed Central PMCID: PMCPMC8678631.
2. Mondal S, Ahlawat S, Koushika SP. Simple microfluidic devices for in vivo imaging of C. elegans, Drosophila and zebrafish. J Vis Exp. 2012;(67). Epub 2012/10/12. doi: 10.3791/3780. PubMed PMID: 23051668; PubMed Central PMCID: PMCPMC3490237.
