# Supplementary material for: Dominant negative variants in KIF5B cause osteogenesis imperfecta via down regulation of mTOR signaling
Source: PLoS Genet. 2023 Nov 7;19(11):e1011005. doi: 10.1371/journal.pgen.1011005 (PMC10656020; doi:10.1371/journal.pgen.1011005)
Supplement: S2 Table — (PDF) [file pgen.1011005.s014.pdf]

S2 Table. ***unc-116*** **genome editing reagents**

crRNA	GCATATGGACAAACATCTTC
T90I repair template	GATGTATTATCCGGTTATAATGGAACAGTTTTTGCATATGGACAAAtATCcagtGGAAAAACACATACAATGGAGgtaggaattatgaaaaccttgataa
T90T repair template	GATGTATTATCCGGTTATAATGGAACAGTTTTTGCATATGGACAAACATCcagtGGAAAAACACATACAATGGAGgtaggaattatgaaaaccttgataa
Primer 6932	ATCCTGCAGGTGCCAATACATTCCTTGTATCCC
Primer 6947	ACCAACGCGTATGGAGAACAACCTTTAGACTAGag
